# Customizing Heterointerfaces in Multilevel Hollow Architecture Constructed by Magnetic Spindle Arrays Using the Polymerizing‐Etching Strategy for Boosting Microwave Absorption

**DOI:** 10.1002/advs.202200804

**Published:** 2022-04-11

**Authors:** Chunyang Xu, Panbo Liu, Zhengchen Wu, Huibin Zhang, Ruixuan Zhang, Chang Zhang, Lei Wang, Longyuan Wang, Bingtong Yang, Ziqi Yang, Wenbin You, Renchao Che

**Affiliations:** ^1^ Laboratory of Advanced Materials Shanghai Key Lab of Molecular Catalysis and Innovative Materials Department of Materials Science Fudan University Shanghai 200438 P. R. China; ^2^ School of Chemistry and Chemical Engineering Northwestern Polytechnical University Xi'an 710129 P. R. China; ^3^ Joint‐Research Center for Computational Materials Zhejiang Laboratory Hangzhou 311100 China

**Keywords:** heterointerfaces, microtube, microwave absorption, multilevel hollow architecture, nanospindle arrays

## Abstract

Heterointerface engineering is evolving as an effective approach to tune electromagnetic functional materials, but the mechanisms of heterointerfaces on microwave absorption (MA) remain unclear. In this work, abundant electromagnetic heterointerfaces are customized in multilevel hollow architecture via a one‐step synergistic polymerizing‐etching strategy. Fe/Fe_3_O_4_@C spindle‐on‐tube structures are transformed from FeOOH@polydopamine precursors by a controllable reduction process. The impressive electromagnetic heterostructures are realized on the Fe/Fe_3_O_4_@C hollow spindle arrays and induce strong interfacial polarization. The highly dispersive Fe/Fe_3_O_4_ nanoparticles within spindles build multi‐dimension magnetic networks, which enhance the interaction with incident microwaves and reinforce magnetic loss capacity. Moreover, the hierarchically hollow structure and electromagnetic synergistic components are conducive to the impedance matching between absorbing materials and air medium. Furthermore, the mechanisms of electromagnetic heterointerfaces on the MA are systematically investigated. Accordingly, the as‐prepared hierarchical Fe/Fe_3_O_4_@C microtubes exhibit remarkable MA performance with a maximum refection loss of −55.4 dB and an absorption bandwidth of 4.2 GHz. Therefore, in this study, the authors not only demonstrate a synergistic strategy to design multilevel hollow architecture, but also provide a fundamental guide in heterointerface engineering of highly efficient electromagnetic functional materials.

## Graphical abstract

A hierarchical multilevel hollow architecture of Fe/Fe_3_O_4_@C microtubes are developed for the first time via a one‐step polymerizing‐etching strategy. The Fe/Fe_3_O_4_@C composites are assembled by hollow spindle arrays and the inner microtube, constructing abundant electromagnetic heterointerfaces and achieving spatially dispersed magnetic nanoparticles, which exhibits strong polarization loss and multi‐scale magnetic loss.

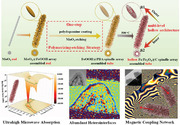

## 1 Introduction

Heterointerface engineering plays a crucial role in tuning conductivity and permittivity of heterogeneous materials, which could induce strong polarization loss and promote microwave absorption (MA).^[^ ^1^, ^2^, ^3^, ^4^, ^5^ ^]^ To utilize heterointerfaces to tailor electromagnetic properties and realize high absorption efficiency, an appropriate composite system should be selected and investigated.^[^ ^6^, ^7^, ^8^, ^9^, ^10^, ^11^, ^12^, ^13^, ^14^ ^]^ However, the precise design and systematic mechanism researches on heterointerfaces for MA have been rarely reported. Fe‐based materials have been regarded as the promising candidates for MA owing to their high conductivity and strong magnetic loss ability.^[^ ^15^, ^16^, ^17^, ^18^, ^19^, ^20^, ^21^, ^22^, ^23^ ^]^ More importantly, Fe‐based materials exhibit multiple phases (Fe_2_O_3_, Fe_3_O_4_, Fe, etc.),^[^ ^24^, ^25^, ^26^, ^27^, ^28^, ^29^ ^]^ and most phase components display remarkable magnetism.^[^ ^29^, ^30^, ^31^, ^32^, ^33^, ^34^, ^35^ ^]^ The multi‐phases transition processes can generate multiple heterogeneous interfaces and can be employed to investigate the microwave attenuation mechanisms of heterointerfaces. To date, less attention has been paid to the heterointerfaces of Fe‐based materials. On the other hand, although Fe‐based absorbers exhibit good MA ability, the high‐density issue and severe aggregation problem severely restrict their practical applications.^[^ ^18^, ^36^ ^]^ Therefore, it is necessary to design a specific morphology to tackle the aggregation issue and reduce density. This can also be used to study the heterointerface effects of Fe‐based microwave absorbers.

Developing hollow structures has been regarded as an effective solution to solve the high‐density issue and produce multiple heterointerfaces.^[^ ^10^, ^37^, ^38^, ^39^, ^40^, ^41^, ^42^, ^43^ ^]^ Constructing the hierarchically hollow architecture can enhance the polarization loss due to the large surface area and increased heterojunctions. Liu et al. fabricated hollow metal@C nanocages with rich heterojunctions, which boosted interfacial polarization and achieved lightweight features.^[^ ^40^ ^]^ Moreover, multilevel hollow structures assembled by the designed nano‐units could increase the number of heterogeneous interfaces and avoid the aggregation of magnetic components,^[^ ^44^ ^]^ promoting interfacial polarization and enhancing magnetic dissipation. For example, the rational design of Fe/C complex materials with hollow structures could create more dielectric/magnetic interfaces and further enhance synergistic effects between magnetic loss and dielectric loss.^[^ ^33^, ^45^, ^46^ ^]^ Li et al. prepared a hollow architecture with a magnetic Fe core, which exhibited strong dielectric/magnetic capacities and offered an optimized impedance balance.^[^ ^47^ ^]^ However, traditional methods to construct hollow structures usually involve time‐consuming and additional etching reagents to remove the templates.^[^ ^48^, ^49^, ^50^ ^]^ And the internal cavity and distribution of heterogeneous interfaces are hard to control in hollow architectures. Therefore, it is necessary to develop a facile and efficient strategy to customize electromagnetic interfaces in hierarchically hollow structures, which plays the fundamental guide in heterointerface engineering for MA applications.

In this study, we demonstrate a novel one‐step polymerizing‐etching strategy to fabricate a multilevel hollow architecture with rich electromagnetic heterointerfaces. A hierarchical microtube constructed by FeOOH@polydopamine (PDA) core‐shell spindle arrays was first prepared using dopamine‐tris‐buffer for the polymerizing‐etching process. Then, a multilevel hollow structure of Fe/Fe_3_O_4_@C was successfully obtained via a controllable reduction process. This synergistic polymerizing‐etching strategy realizes the tubular structure using MoO_3_ template without using additional alkaline agents, such as ammonia. The unique hollow “spindle‐on‐tube” structure endows absorbents with lightweight features and solves the aggregation issue of magnetic particles. Compared with the traditional Fe‐based absorbents, the as‐prepared Fe/Fe_3_O_4_@C tubular heterostructures exhibit strong polarization loss induced by the large hollow structure and abundant heterointerfaces, as confirmed by the high‐resolution transmission electron microscopy (HRTEM) images and electron holography. Furthermore, highly dispersive Fe/Fe_3_O_4_ particles suspended in this hierarchical microtube build multi‐scale magnetic coupling networks, which enhance the interaction with incident microwaves and strengthen magnetic loss capacity. Accordingly, Fe/Fe_3_O_4_@C microtubes exhibit superior MA performance with a maximum refection loss value of −55.4 dB and an effective absorption bandwidth of 4.2 GHz. Therefore, in this study, we not only present a facile method to prepare a multilevel hollow structure, but also provide a fundamental guide in heterointerface engineering for developing highly efficient microwave absorbers.

## 2 Results and Discussion

**Figure 1:**
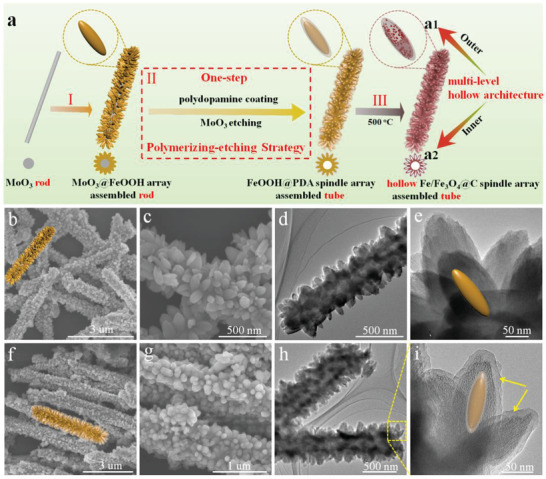
a) Schematic process of constructing multilevel hollow architecture of Fe/Fe_3_O_4_@C composites with hollow nanospindle arrays via the polymerizing‐etching strategy. a1) outer and a2) inter hollow units. b,c) SEM and d,e) TEM images of MoO_3_@FeOOH array assembled rods. f,g) SEM and h,i) TEM images of FeOOH@PDA array assembled tubes.

The synthetic processes for fabricating multilevel hollow architecture of Fe/Fe_3_O_4_@C microtubes with hollow spindle arrays are illustrated in **Figure** **1**. First, uniform MoO_3_ rods were synthesized using a hydrothermal method.^[^ ^51^ ^]^ As shown in scanning electron microscopy (SEM) images (Figure S1a,b, Supporting Information), the MoO_3_ samples have a rod‐like shape with an average width of ≈400 nm. The X‐ray powder diffraction (XRD) pattern indicates that MoO_3_ (JCPDS No. 47‐1320) are synthesized successfully (Figure S1c, Supporting Information). Second, the spindle‐like FeOOH arrays were uniformly grown on MoO_3_ rods via a facile reflux process to obtain the MoO_3_@FeOOH core‐shell rod (Figure 1a). As shown in Figure 1b,c, high‐density FeOOH nanospindles are anchored on the MoO_3_ rod, forming a unique spindle‐array assembled rod with high dipolar distribution. Transmission electron microscopy (TEM) images further confirm that the surface of MoO_3_ is uniformly decorated with spindle arrays. The XRD pattern reveals that all the peaks are corresponded to the crystalline FeOOH (JCPDS no. 75‐1594), except some peaks from MoO_3_ at 12.84°, 23.67°, and 25.83°, which further proves the MoO_3_@FeOOH core‐shell structure (as shown in Figure S2, Supporting Information).

**Figure 2:**
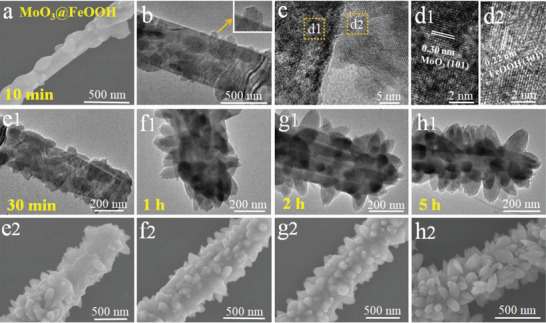
Time‐dependent structure evolution of MoO_3_@FeOOH array assembled rods: a) SEM, b,c) TEM, and d) HRTEM images of MoO_3_@FeOOH core‐shell composites prepared within 10 min. TEM and SEM images of MoO_3_@FeOOH core‐shell composites prepared within: e1,e2) 30 min, f1,f2) 1 h, g1,g2) 2 h, and h1,h2) 5 h.

Such high‐quality spindle arrays provide a new way for hierarchical microstructure engineering. To illustrate the formation of MoO_3_@FeOOH core‐shell rods, we demonstrate the evolution of a time‐dependent structure. First, after mixing MoO_3_ and the FeCl_3_ solution for 10 min, few FeOOH nanoparticles were deposited on the MoO_3_ rod, and HRTEM images verify this core‐shell structure (**Figure** **2**a–d). By prolonging the reaction time from 30 min to 2 h, more FeOOH nanoparticles were grown on the surface of the MoO_3_ rod, and nanoparticles were growing in the shape of spindles (Figure 2e–g). Furthermore, MoO_3_ rods were fully covered with spindle‐shaped FeOOH in 5 h, forming a core‐shell “spindle‐on‐rod” structure. Such unique core‐shell morphology plays a vital role in constructing the final multilevel hollow architecture.

Subsequently, using a polymerizing‐etching strategy, FeOOH nanospindles were coated with PDA (FeOOH@PDA) and the MoO_3_ core was etched during the dopamine polymerizing process. It is noteworthy that no additional agents, such as ammonia, were used for creating the alkaline environment in our strategy to remove the inner MoO_3_ because the tris‐buffer solution can etch the MoO_3_ rod and produce the inner tubular structure. After dopamine polymerization at room temperature, a smooth PDA layer was coated on the surface of FeOOH nanospindles (Figure 1f,g). TEM images confirm that the PDA layer is successfully grown on FeOOH and the thickness of the PDA layer is 20 nm (marked with yellow arrows in Figure 1i. The XRD result shows that all the diffraction peaks can be assigned to FeOOH, and the MoO_3_ core is removed during the polymerization process of dopamine (as displayed in Figure S3, Supporting Information). Therefore, the unique hierarchical microtubes constructed by ultrahigh‐density FeOOH@PDA core‐shell arrays are obtained via a polymerizing‐etching strategy by using the MoO_3_ rod as a sacrificial template.

**Figure 3:**
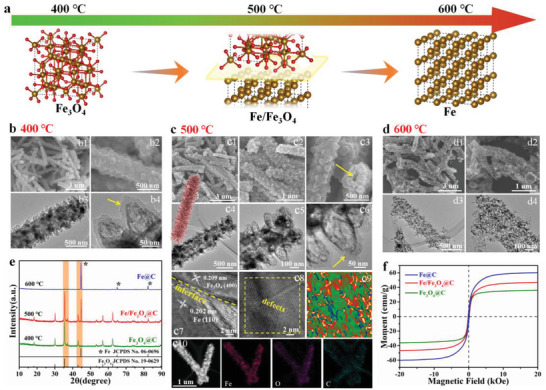
a) Formation scheme for controllable magnetic components and heterointerfaces of Fe_3_O_4_@C, Fe/Fe_3_O_4_@C, and Fe@C tubular heterostructures and corresponding multilevel hollow architecture at b) 400, c) 500, and d) 600 °C. b1‐b2, c1‐c3, d1‐d2) SEM images and b3‐b4, c4‐c6, d3‐d4) TEM images for the composites at 400 °C, 500 °C and 600 °C. e) XRD patterns and f) hysteresis loops of as‐prepared Fe_3_O_4_@C, Fe/Fe_3_O_4_@C, and Fe@C composites.

By carefully annealing in Ar/H_2_ atmosphere, the FeOOH@PDA core‐shell composite can be converted to the metal@C heterostructure, constructing multilevel hollow architecture with magnetic/dielectric configurations. As illustrated in **Figure** **3**a, FeOOH components can be transformed into the Fe_3_O_4_ phase, binary Fe/Fe_3_O_4_, and Fe phase at 400, 500, and 600 °C, respectively. Particularly, rich magnetic heterointerfaces and dielectric/magnetic interfaces can be generated at 500 °C. As displayed in XRD results (Figure 3e), the FeOOH core is converted into the Fe_3_O_4_ phase (JCPDS no. 19‐0629) at 400 °C. Moreover, the PDA shell could be transformed into carbon layers. Then, Fe_3_O_4_@C composites are obtained at 400 °C. SEM images (Figure 3b) reveal that as‐prepared Fe_3_O_4_@C samples maintain the hierarchical microtube structure, but the solid core‐shell FeOOH@PDA was transformed into hollow Fe_3_O_4_@C spindles, which can be clearly observed in TEM images. When FeOOH@PDA composites were annealed at 500 °C, new diffraction peaks of Fe (JCPDS no. 06‐0696) are produced (Figure 3e), and ternary Fe/Fe_3_O_4_@C composites are obtained. The microstructure of Fe/Fe_3_O_4_@C composites is further characterized by SEM and TEM images. As shown in Figure 3c, Fe/Fe_3_O_4_@C microtubes obtained at 500 °C retain the structure of the hierarchical tube assembled by spindle arrays. Moreover, the presence of the inner tube was proved by the cavity, as marked with the yellow arrow (Figure 3c3). Surprisingly, the solid core‐shell FeOOH@PDA spindles are converted into the hollow Fe/Fe_3_O_4_@PDA spindles, as observed in TEM images (Figure 3c4–c6). Therefore, a multilevel hollow architecture is successfully constructed, which is composed of hollow spindles array and the inner microtube (Figure 1a1,a2). In this unique hollow nanostructure, magnetic nanoparticles (Fe and Fe_3_O_4_) are suspended within the spindle‐shaped carbon shell. Owing to the phase transition, ternary Fe/Fe_3_O_4_@C composites could exhibit the rich hetero‐interfaces property, which is proved by the HRTEM images. In Figure 3c7, a lattice fringe exhibits the interlayer spacing of 0.209 nm corresponding to the (400) crystal plane of Fe_3_O_4_. Moreover, another interplanar spacing of 0.28 nm is assigned to the (110) lattice fringe of Fe. Therefore, a heterogeneous interface (Figure 3g), marked with a yellow dashed line) is formed between Fe_3_O_4_ and Fe phases. Moreover, abundant defects can be observed in the entire crystal (Figure 3c8). Using geometric phase analysis, the corresponding strain field micrograph map is shown in Figure 3c9. Numerous spots of varying brightness owing to abundant defects are observed in these hierarchical tubular heterostructures. These defects can contribute to interfacial polarization and consume microwave energy. Elemental mapping images from energy‐dispersive X‐ray spectroscopy show that Fe, O, and C elements are uniformly distributed in the Fe/Fe_3_O_4_@C composite. However, when the annealing temperature was increased to 600 °C, all the diffraction peaks were assigned to Fe, indicating that FeOOH@PDA composites were completely converted to Fe@C nanospindles (Figure 3e). As displayed in Figure 3d, after annealed at 600 °C the architecture of spindle‐assembled microtube was hardly retained and many cuboid particles were produced on the surface owing to the phase transition from Fe_3_O_4_ to Fe. The XRD, SEM, and TEM results reveal that FeOOH@PDA solid spindle‐assembled microtubes successfully transformed into Fe_3_O_4_@C, Fe/Fe_3_O_4_@C, and Fe@C hollow spindle‐assembled microtubes, respectively. Such magnetic components dispersed in this “spindle‐on‐tube” structure can result in higher saturation magnetization (Ms). As displayed in Figure 3f, the Ms values of Fe_3_O_4_@C, Fe/Fe_3_O_4_@C, and Fe@C are 36.14, 46.84, and 60.21 emu g^−1^. High Ms values of these unique multilevel hollow architecture could enhance magnetic storage and strengthen magnetic loss ability,^[^ ^19^, ^44^ ^]^ further boosting MA performance.

**Figure 4:**
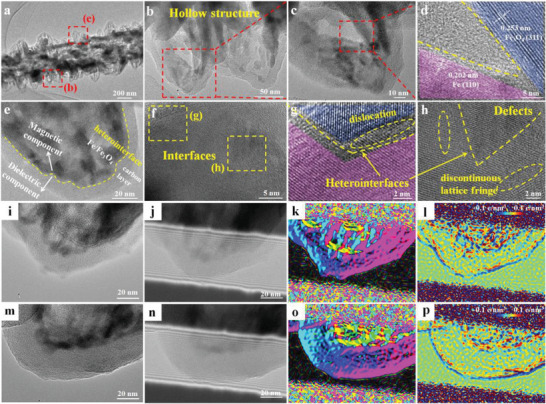
Multilevel hollow architecture of Fe/Fe_3_O_4_@C tubular heterostructures: a–c,e–f) TEM images, and d,g,h) HRTEM images with obvious heterointerfaces and defects. i,m) TEM images, j–l,n–p) corresponding off‐axis electron holograms and reconstructed holograms images of Fe/Fe_3_O_4_@C composites for charge density.

As‐prepared Fe/Fe_3_O_4_@C tubular heterostructures composed of abundant hollow spindle arrays provide a large surface area and abundant heterointerfaces, which are beneficial to inducing polarization loss. Generally, there are various kinds of polarization loss mechanisms and interfacial polarization usually occurs on the heterogeneous interfaces due to different dielectric properties and electrical conductivities of components. To prove that abundant heterointerfaces are effectively customized in our multilevel hollow architecture, the microstructures of Fe/Fe_3_O_4_@C microtubes were carefully investigated through TEM images. As displayed in **Figure** **4**a–d, the Fe/Fe_3_O_4_@C microtube is made up of multilevel hollow units, that is, the outer hollow spindles and inner tubes. Moreover, the obvious lattice fringes of Fe and Fe_3_O_4_ can be observed clearly, which constitutes magnetic heterointerfaces. As shown in Figure 4e, a single nanospindle is composed of carbon layers and Fe/Fe_3_O_4_ nanoparticles, and the large magnetic/dielectric heterointerfaces are produced in spindle arrays. Among the internal nanoparticles, magnetic heterointerfaces and defects can be found easily. Such heterointerfaces can serve as polarization centers and trigger stronger polarization loss, thereby boosting MA performance. To further prove the multiple heterointerfaces in this multilevel hollow composite, TEM electron holography technology was used to reveal the different contacting interfaces and related interfacial polarization.^[^ ^22^, ^44^ ^]^ As shown in Figures 4i–p,6b, the spatial distribution of charge is clearly observed in reconstructed phase images, where negatives charges are represented by blue areas. In C‐Fe/Fe_3_O_4_ and Fe‐Fe_3_O_4_ interfaces, positive and negative charges are gathered at the two sides of these contacting interfaces, building the spatial electric field. When the incident microwave is applied on these interfaces, the electronic migration can cause intensive interfacial polarization and relaxation, converting electromagnetic wave energy into thermal energy. Therefore, abundant heterointerfaces were successfully customized in Fe/Fe_3_O_4_@C hierarchical microtubes, and the induced interfacial polarization promoted MA performance.

The Raman spectra are further employed to analyze the structural defects of as‐prepared samples (Figure S4, Supporting Information). The value of *I*_D_/*I*_G_ increased with high temperature, and the Fe@C composites exhibit the highest *I*_D_/*I*_G_ value of 1.16 owing to the excess defects produced at 600 °C. This is consistent with the specific morphology change. Chemical valence states of Fe/Fe_3_O_4_@C composites are characterized by the X‐ray photoelectron spectroscopy (XPS) technique. As shown in Figure S5a, Supporting Information, the Fe 2p spectrum can be divided into two peaks of 708.85 eV for Fe^2+^ 2p_3/2_ and 722.31 eV for Fe^2+^ 2p_1/2_ and two peaks of 710.89 and 725.08 eV for Fe^3+^ 2p_3/2_ and 2p_1/2_, respectively.^[^ ^19^, ^34^ ^]^ Typically, the Fe–O bond is confirmed by the peak of 528.61 eV in the O 1s spectrum (Figure S5b, Supporting Information). The C 1s spectrum can be divided into three parts. The peaks at 286.87 and 284.34 eV correspond to C═O and C–N bonds, respectively, whereas the peak at 282.96 eV relates to C–C/C═C.^[^ ^19^ ^]^ Remarkably, as‐prepared Fe/Fe_3_O_4_@C tubular heterostructures can be considered as a magnetic network and a distinct conductive structure, thus displaying the great potential to achieve superior MA performance.

**Figure 5:**
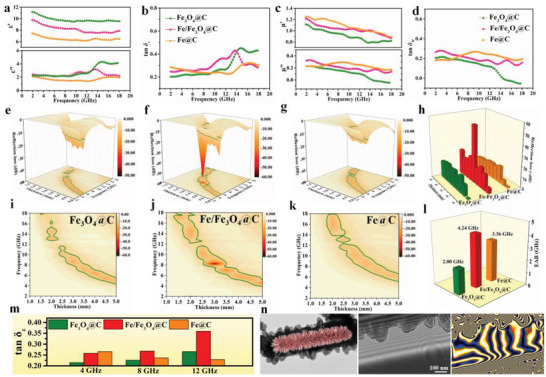
a, c) Electromagnetic parameters, b) dielectric loss tangent, and d) magnetic loss tangent of Fe_3_O_4_@C, Fe/Fe_3_O_4_@C, and Fe@C composites. 3D plots of RL of e) Fe_3_O_4_@C, f) Fe/Fe_3_O_4_@C, and g) Fe@C composites, and h) 3D bars of RL for three samples. The reflection loss values and effective absorption bandwidth of i) Fe_3_O_4_@C, j) Fe/Fe_3_O_4_@C, and k) Fe@C samples, and l) corresponding comparison of effective absorption bandwidth. m) Comparison of dielectric loss tangent and n) TEM image, corresponding off‐axis electron holograms and reconstructed hologram images of Fe/Fe_3_O_4_@C composites for magnetic field lines.

Electromagnetic parameters of as‐prepared Fe_3_O_4_@C, Fe/Fe_3_O_4_@C, and Fe@C microtubes are investigated to reveal the impacts of composition and microstructure on MA performance. Generally, the real parts of complex permittivity and permeability (*ε*′ and *µ*′, respectively) imply the ability of storing electromagnetic energy, whereas the imaginary parts (*ε*″ and *µ*″, respectively) suggest the capability to dissipate electromagnetic energy. It was noted that *ε*' and *ε*" decrease with the increase in temperature from 400 to 600 °C (**Figure** **5**a). Higher *ε*′ values suggest better conductivity, whereas the decrease in *ε*″ means that dielectric loss capability is declining. To further evaluate the dielectric loss property, the dielectric loss tangent *δε* (tan *δε* = *ε*″/*ε*′) is calculated. The tan *δε* of Fe/Fe_3_O_4_@C composite is larger than that of the other two samples in a wider frequency range, which indicates the enhanced dielectric loss abilities resulting from the multiple heterointerfaces, rich defects, better conductivity, and multilevel hollow structure. Contrariwise, the value of *µ*′ increases with the annealing temperature, suggesting magnetic response is increased with temperature. The tan *δ*_M_ of Fe/Fe_3_O_4_@C composite remains high, demonstrating the strong magnetic loss capacity owing to the Fe/Fe_3_O_4_ component and multi‐scale magnetic network originating from the hierarchical microtube. Based on the abovementioned electromagnetic parameters, Fe/Fe_3_O_4_@C microtubes are expected to display higher MA performance. As‐prepared Fe/Fe_3_O_4_@C microtubes can exhibit strong dielectric dissipation owing to hollow structures and rich heterointerfaces, and remarkable magnetic loss abilities due to the highly dispersive Fe/Fe_3_O_4_ nanoparticles within spindle arrays.

To evaluate the dissipation property of as‐synthesized Fe_3_O_4_@C, Fe/Fe_3_O_4_@C, and Fe@C composites toward the incident microwave, the 3D plots of reflection loss (RL) values for different thickness are displayed in Figure 5e–g. The Fe_3_O_4_@C composite exhibits the middle capability of MA with RL = −18.1 dB for the thickness of 4 mm. For binary magnetic/dielectric composites, the Fe/Fe_3_O_4_@C sample demonstrates a remarkable MA performance with the highest maximum RL value of −55.4 dB at the thickness of 3 mm. Such superior MA ability results from the synergetic components of magnetic Fe/Fe_3_O_4_ and dielectric carbon layers and the enhanced multiple interface‐induced polarization loss. However, the Fe@C composite obtained at 600 °C exhibit poor MA capability with a maximum RL value of only −15.2 dB. Although the Fe@C composite displays strong magnetic response with high *µ*′ and *µ*″ values, the dielectric loss ability is poor and not conducive to the dissipation of electromagnetic energy. Moreover, Fe/Fe_3_O_4_@C microtubes also exhibit a wider absorption bandwidth of 4.24 GHz, which is much larger than that of Fe_3_O_4_@C and Fe@C (Figure 5i,l). It is worth noting that absorption ability and bandwidth can be efficiently adjusted by engineering heterointerfaces, hollow structure, and multiple components.

**Figure 6:**
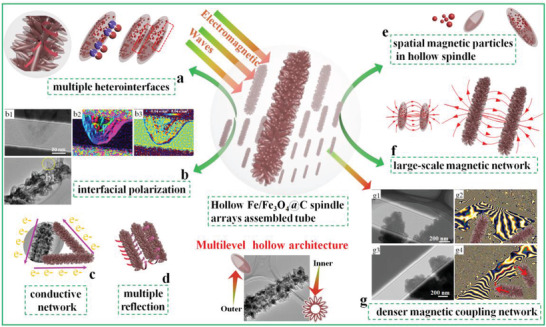
MA mechanism in the hierarchical Fe/Fe_3_O_4_@C multilevel hollow architecture. a,b) the multiple heterointerfaces and ralated interfacial polarization. c) conductive networks. d) multiple reflection. e, f) the multi‐scale magnetic network and g) related electron holography images.

Compared with the reported metal–carbon MA materials, as‐synthesized Fe/Fe_3_O_4_@C microtubes demonstrate remarkable MA performance (as shown in Table S1, Supporting Information). Accordingly, the rational construction of multilevel hollow architecture, engineering abundant heterointerfaces, the combination of Fe/Fe_3_O_4_ magnetic components and dielectric carbon layer contribute to the electromagnetic energy storage/conversion and boost MA performance. The associated electromagnetic energy absorption/conversion mechanisms can be illustrated as following aspects (**Figure** **6**).

### 2.1 Polarization Loss by Abundant Heterointerfaces

Fe/Fe_3_O_4_@C hierarchical tubular composites possess abundant heterointerfaces (Figures 3,4), which is of great benefit to the interfacial polarization behavior and dielectric loss capacity. The hierarchical Fe/Fe_3_O_4_@C composites are composed of dielectric carbon shell and magnetic Fe/Fe_3_O_4_ nanoparticles. In this unique “spindle‐on‐tube” architecture, there are multiple kinds of heterointerfaces, including Fe/Fe_3_O_4_ interface, Fe/Fe_3_O_4_‐C interface, and adjacent spindle‐shape shell interface (Figures 4,6a). The electron holograms of Fe/Fe_3_O_4_@C composite reveal a large number of positive charge and negative electrons are distributed around the interfacial region of Fe/Fe_3_O_4_@C nanospindles (Figures 4i–p,6b). The unbalanced charges gather around heterogeneous interfaces when the microwave propagates through these interfaces. The electrons transfer to the heterointerfaces and cause intensive interfacial polarization and dielectric relaxation, facilitating the conversion of microwave energy into thermal energy. Besides, interfacial polarization loss will also occur on porous or hollow materials due to the different dielectric properties between materials and air medium. In this multilevel hollow structure, large surface areas with heterogeneous interfaces are expected to induce strong interfacial polarization and further enhance the microwave absorbing performance.

Additionally, numerous carbon–heteroatoms groups (C–O and C–N; Figure S5, Supporting Information) and defects (Figures 3c,4g,h) in Fe/Fe_3_O_4_@C composite can act as dipole sites. Related dipole polarization and defect‐induced polarization loss can also contribute to higher MA performance. Moreover, carbon spindle arrays can be regarded as a 1D conductive network (Figure 6c). Fe/Fe_3_O_4_@C microtubes exhibit high permittivity, and various electron transportation channels are formed based on these spindles and microtubes. Conduction loss ability can be promoted by the formed 1D conductive network, which contributes to high MA performance.

### 2.2 Natural Resonance and Magnetic Coupling Network by Hollow Spindle Arrays

Usually, the magnetic loss is defined as four main mechanisms: magnetic hysteresis, domain‐wall resonance, natural resonance, and eddy current effect.^[^ ^52^, ^53^, ^54^ ^]^ The first two mechanisms can be excluded in the weak electromagnetic field and gigahertz range. For natural resonance, it describes the energy absorption of ferromagnetic materials with large magnetization. Typically, some magnetic materials with strong anisotropy will display higher natural resonance frequencies. In this work, the Fe/Fe_3_O_4_@C hierarchical microtubes exhibit strong natural resonance due to its higher saturation magnetization and considerable anisotropy originated from 1D spindle arrays. As for eddy current effect, the value of *C*_0_ (*C_0_ = µ*″*(µ′)^−^* ^2^ *f ^−^* ^1^) gradually decreases over the frequency (Figure S6, Supporting Information), which suggests the eddy current loss is not the main loss mechanism for the energy absorption. In conclusion, in this work the magnetic loss is mainly derived from the natural resonance.

The unexpected self‐assembled aggregation of magnetic nanoparticles cannot be avoided easily owing to the interacted attraction effect. Furthermore, after being incorporated with dielectric ingredients, magnetic loss capacity of the composite might be weakened compared to that of the original magnetic nanoparticles. Using a polymerizing‐etching strategy and controllable reduction process, the as‐synthesized Fe/Fe_3_O_4_@C spindle arrays can not only solve the aggregation of magnetic components but also achieve a much higher loading ratio of magnetic nanoparticles. At nano‐micro level, our tubular architecture assembled by nanospindle arrays provided a robust matrix to support and disperse the abundant suspended Fe/Fe_3_O_4_ nanoparticles, thereby solving the aggregation issue (Figures 3,6e). A unique multi‐scale magnetic Fe/Fe_3_O_4_@C network is assembled by abundant nanospindle arrays, which contributes to high magnetic permeability and loss abilities (Figures 3c,6f,g; Figure S6, Supporting Information), as confirmed by electron holography.^[^ ^34^, ^44^ ^]^ Even though the Fe/Fe_3_O_4_ nanoparticles are wrapped in a PDA‐derived carbon shell, denser magnetic field lines can still penetrate through the non‐magnetic ingredient surface (Figure S7, Supporting Information). Additionally, the top and side surfaces of the assembled tube display high‐density magnetic flux lines (Figure 6g1,g2), which expands the magnetic responding area far beyond the size. Moreover, the neighboring nanospindles from two microtubes exhibit remarkable magnetic coupling, which was verified by the integral magnetic field lines among the samples (Figure 6g3,g4). The magnetic network region is enlarged to the millimeter scale to meet the length of the microwave and further reinforces the magnetic dissipation abilities. Furthermore, the magnetization can be effectively strengthened by the high loading ratio of Fe/Fe_3_O_4_ nanoparticles embedded in these ultrahigh‐density spindle arrays (Figure 3c,6e,g). This can be confirmed by the strong Ms and high permeability (Figure 3f,5c,d). Therefore, compared to the traditional magnetic composites, the developed Fe/Fe_3_O_4_@C hierarchical tubular composites realize a highly distributed magnetic network and ultrahigh‐density loading of magnetic components, which exhibits remarkable magnetic attenuating ability.

The micromagnetic simulation is employed to reveal the abovementioned coupling network in this ultrahigh‐density array. As shown in Figure S8, Supporting Information, the magnetic moments in two adjacent nanospindles move along the same direction, indicating a remarkable magnetic coupling.^[^ ^44^ ^]^ Meanwhile, the cross‐sectional images in cyclical variation of magnetic moments of this unique nanospindle array reveal that magnetic moments could move faster in a single period with the increasing Fe component (from Figure S8b–e, Supporting Information), suggesting that the introduction of binary Fe/Fe_3_O_4_ components can contribute to the enhanced magnetic loss effect of the domain wall migration. Therefore, high loading of magnetic Fe/Fe_3_O_4_ components can accelerate the movement of magnetic moments and enhance magnetic loss ability.

### 2.3 Hierarchical 1D Tubular Structure and Synergic Magnetic/Dielectric System

Benefitting from the unique multi‐scale hierarchically hollow structure, the exposed large surface areas of the Fe/Fe_3_O_4_@C microtube could produce multi‐reflection and multi‐scattering to dissipate microwave energy (Figure 6d). Abundance of hollow spindles and hierarchical microtubes are conducive to generate large interior cavities and interspace (Figures 3,4). This hierarchically hollow materials are conducive to the impedance matching between absorbing materials and air medium. Furthermore, the assembled composite is made up of magnetic Fe/Fe_3_O_4_ and dielectric carbon layers. Fe/Fe_3_O_4_@C composites can effectively attenuate microwave energy through strong magnetic loss and remarkable dielectric loss in this system compared to either single carbon materials or individual Fe‐based materials. Electromagnetic synergistic components are also in favor of impedance matching. Relying on the aforementioned advantages of hierarchical tubular structure and multiple absorption mechanism, the Fe/Fe_3_O_4_@C composites demonstrate remarkable MA performance that surpasses a majority of Fe‐based absorbents and other magnetic materials (as shown in Table S1, Supporting Information).

## 3 Conclusion

We have established a polymerizing‐etching strategy to customize abundant heterointerfaces in multilevel hollow architecture with tunable components (Fe_3_O_4_@C, Fe/Fe_3_O_4_@C, and Fe@C). The core‐shell FeOOH@PDA nanospindle arrays were prepared first and then converted into hollow magnetic metal@C spindles assembled on a hierarchical microtube. This unique “nanospindles on microtube” structure is composed of rich electromagnetic heterointerfaces, which could induce strong interfacial polarization loss. Moreover, the aggregation issue of Fe/Fe_3_O_4_ particles can be avoided effectively by this multilevel hollow architecture, forming a multi‐scale magnetic coupling network. Owing to the hierarchical hollow structure, rich electromagnetic heterointerfaces, highly dispersive magnetic particles, and 1D carbon microtube, as‐prepared Fe/Fe_3_O_4_@C microtubes exhibited superior MA performance with a maximum refection loss value of −55.4 dB and effective absorption bandwidth of 4.2 GHz. We believe that our polymerizing‐etching strategy will open new doors to engineer heterointerfaces and develop hollow structures for high‐performance microwave absorbers.

## 4 Experimental Section

All chemicals used were of analytical grade and were used directly without further purification.

### Synthesis of MoO_3_

MoO_3_ nanorods were prepared according to the previous work. In a typical hydrothermal process, 0.5793 g ammonium molybdate tetrahydrate and 2.5 mL of HNO_3_ were first dissolved in 30 mL of deionized water. After stirring for 10 min, the solution was transferred into a Teflon‐lined stainless autoclave (50 mL), which was kept at 180 °C for 12 h. Finally, the products were washed repeatedly with deionized water for several times before drying at 70 °C.

### Synthesis of MoO_3_@FeOOH

The MoO_3_@FeOOH sample was prepared by a facile refluxing method. 30 mg of MoO_3_ was first dissolved in 60 mL deionized water to get a white solution. Then 1.95 g of FeCl_3_ was added. The solution was kept stirring in an oil bath at 80 °C for 5 h. The final products were collected and washed with deionized water and dried at 70 °C.

### Synthesis of FeOOH@PDA

First, 60 mg of MoO_3_@FeOOH and 120 mg tris‐buffer were dissolved in 100 mL deionized water. Then 120 mg of C_8_H_11_NO_2_·HCl was added under stirring and kept stirring for 2 h. The precipitate was washed with deionized water and dried at 70 °C.

### Synthesis of Fe_3_O_4_@C, Fe/Fe_3_O_4_@C, and Fe@C

In a typical synthesis, as‐prepared FeOOH@PDA were placed in a quartz boat under an H_2_/N_2_ (5% H_2_) atmosphere and the furnace was heated to 400, 500, and 600 °C at a rate of 2 °C min^−1^ for 4 h to obtain Fe_3_O_4_@C, Fe/Fe_3_O_4_@C, and Fe@C, respectively.

### Microwave Absorption Measurements

The measured samples were first prepared by adding the absorbents (50 wt%) into molten paraffin and uniformly mixing them, followed by modeling into a coaxial ring with the outer diameter of 7.0 mm and inner diameter of 3.0 mm. The experimental set‐up for preparing coaxial ring samples is displayed in Figure S9, Supporting Information. Electromagnetic parameters (complex permittivity and complex permeability) were measured by a N5230C vector network analyzer over the range of 2–18 GHz. The RL values were calculated based on the transmission line theory:

(1) $$Z_{\mathrm{in}}=\sqrt{\mu_{r}/\varepsilon_{r}}\tan\;h\left|-j\left(2\pi fc\right)\sqrt{\varepsilon_{r}\mu_{r}}\right|$$

(2) $$RL\left(\mathrm{dB}\right)=-20\log\left|Z_{\mathrm{in}}-1/Z_{\mathrm{in}}+1\right|$$

where *ε*_r_ and *μ*_r_ are the complex permittivity (*ε*_r_ *= ε*′ − j*ε′′*) and permeability (*μ*_r_ *= μ′ − jμ′′*), respectively, *f* is the frequency of microwave, *c* is the velocity of light, *d* is the thickness, and *Z*_in_ is the normalized input impedance of the sample.

### Characterizations

The crystalline phase and purity of the products were analyzed by powder XRD (Bruker, D8‐Advance X‐ray diffractometer, Germany) using Ni‐filtered Cu Ka radiation. The morphology and structure of the products were examined by a field‐emission SEM on a Hitachi S‐4800 with an accelerating voltage of 5 kV and a field‐emission TEM (JEOL, JEM‐2100F, 200 kV). The Raman spectra were acquired with a Renishaw Invia spectrometer using a 514 nm laser excitation. XPS spectra were obtained on an ESCALab MKII X‐ray photoelectron spectrometer using Al K*α* X‐ray as the excitation source. The hysteresis loops were performed with a superconducting quantum interference device (MPMS(SQUID) VSM) magnetometer (Quantum Design Company).

### Statistical Analysis

Data in Figures 4k–l,o–p,6b,g were obtained by the reconstruction of electronic holography images, using Gatan Microscopy Suite software. The related information of electronic holography technology is displayed in Figure S10, Supporting Information.

## Conflict of Interest

The authors declare no conflict of interest.

## Supporting information

Supporting Information

## Data Availability

The data that support the findings of this study are available from the corresponding author upon reasonable request
