# Supplementary material for: Customizing Heterointerfaces in Multilevel Hollow Architecture Constructed by Magnetic Spindle Arrays Using the Polymerizing‐Etching Strategy for Boosting Microwave Absorption
Source: Adv Sci (Weinh). 2022 Apr 11;9(17):2200804. doi: 10.1002/advs.202200804 (PMC9189646; doi:10.1002/advs.202200804)
Supplement: Supplementary file 1 — Supporting Information [file ADVS-9-2200804-s001.pdf]

ADVANCED SCIENCE
Open Access

Supporting Information

for *Adv. Sci.*, DOI 10.1002/advs.202200804

Customizing Heterointerfaces in Multilevel Hollow Architecture Constructed by Magnetic Spindle Arrays Using the Polymerizing-Etching Strategy for Boosting Microwave Absorption

*Chunyang Xu, Panbo Liu, Zhengchen Wu, Huibin Zhang, Ruixuan Zhang, Chang Zhang, Lei Wang, Longyuan Wang, Bingtong Yang, Ziqi Yang, Wenbin You and Renchao Che**

Supporting Information

Customizing heterointerfaces in multilevel hollow architecture constructed by magnetic spindle arrays using polymerizing-etching strategy for boosting microwave absorption

*Chunyang Xu, Panbo Liu, Zhengchen Wu, Huibin Zhang, Ruixuan Zhang, Chang Zhang, Lei Wang, Longyuan Wang, Bingtong Yang, Ziqi Yang, Wenbin You, Renchao Che**

C. Y. Xu, Z. C. Wu, H. B. Zhang, R. X. Zhang, C. Zhang, L. Wang, L. Y. Wang, B. T. Yang, Z. Q. Yang, W. B. You, Prof. R. C. Che

Laboratory of Advanced Materials, Shanghai Key Lab of Molecular Catalysis and Innovative Materials, Department of Materials Science, Fudan University, Shanghai 200438, P.R. China, E-mail: rcche@fudan.edu.cn

Prof. R. C. Che

Joint-Research Center for Computational Materials, Zhejiang Laboratory, Hangzhou 311100, China

Prof. P. B. Liu

School of Chemistry and Chemical Engineering, Northwestern Polytechnical University, Xi'an 710129, P. R. China

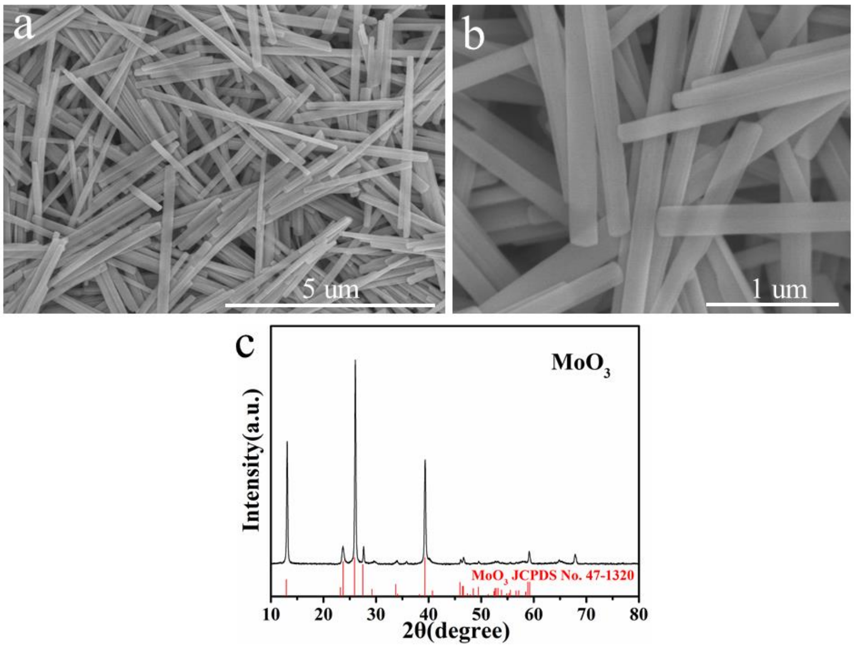

Figure S1. a-b) SEM images and c) XRD pattern of MoO_3 samples.

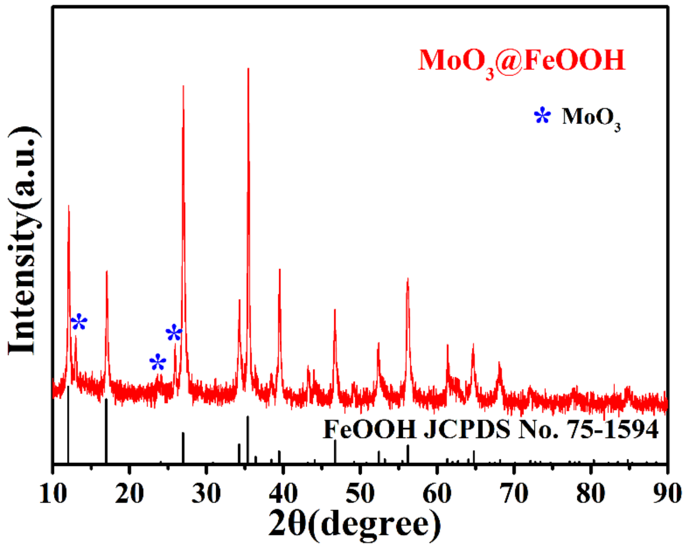

Figure S2. XRD patterns of MoO_3@FeOOH rod.

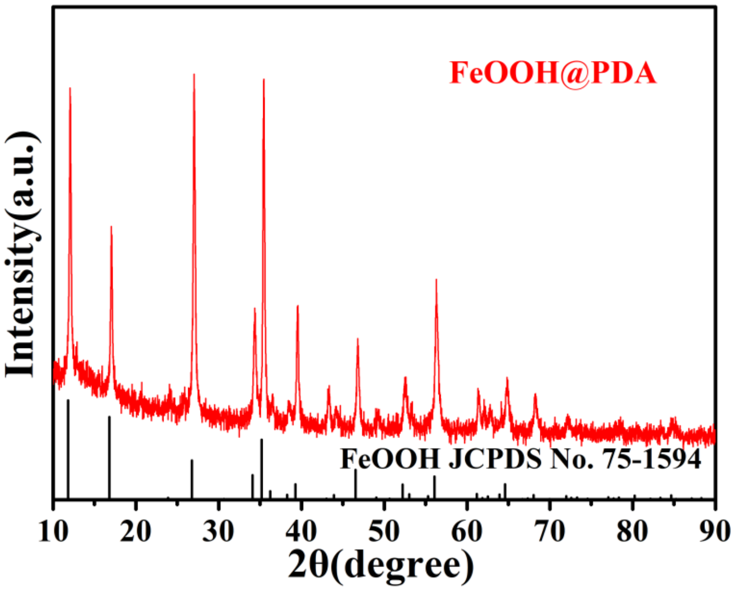

Figure S3. XRD patterns of FeOOH@PDA samples.

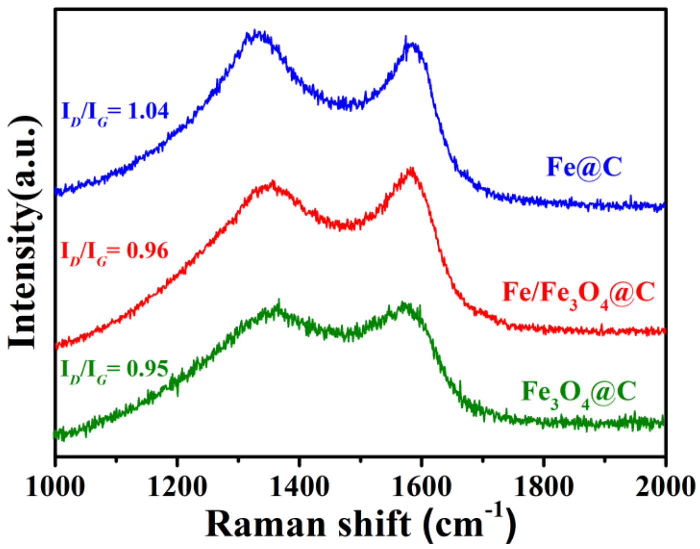

Figure S4. Raman spectra as-prepared Fe_3O_4@C, Fe/Fe_3O_4@C and Fe@C composites.

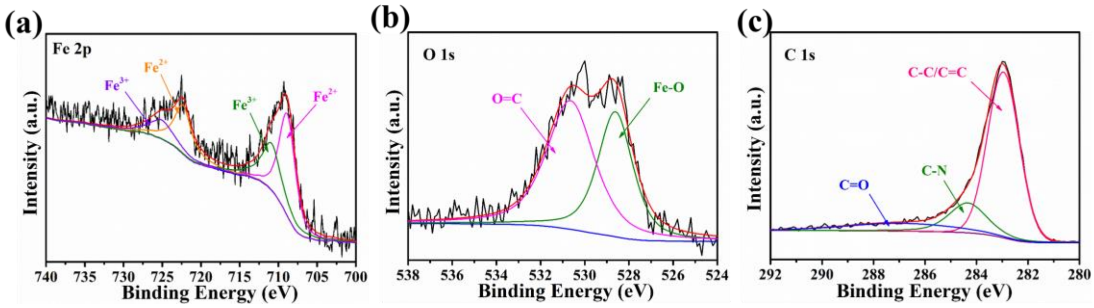

Figure S5. High resolution XPS spectra of d) Fe 2p, e) O 1s and f) C 1s for $Fe/Fe_3O_4@C$ composite.

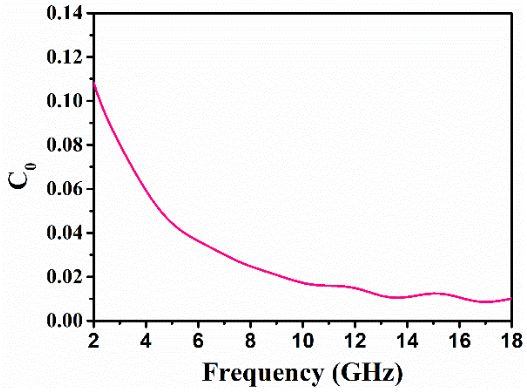

Figure S6. The C_0 value of as-prepared $Fe/Fe_3O_4@C$ composites.

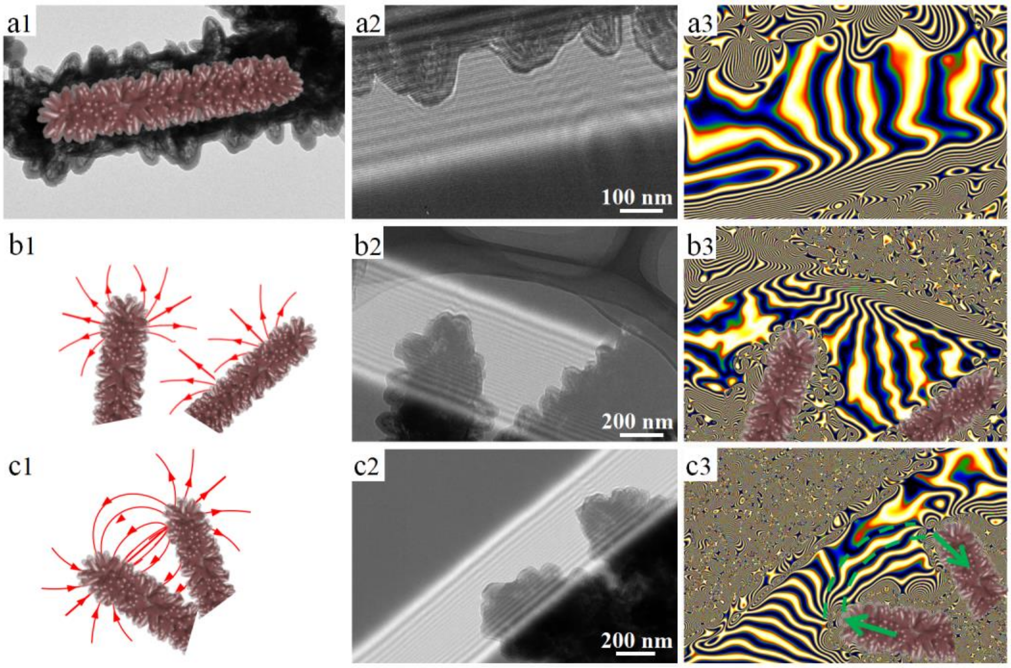

Figure S7. a1) TEM images, b1-c1) illustration of magnetic field lines and a2-a3,

b2-b3, c2-c3) corresponding off-axis electron holograms of $Fe/Fe_3O_4@C$ composites.

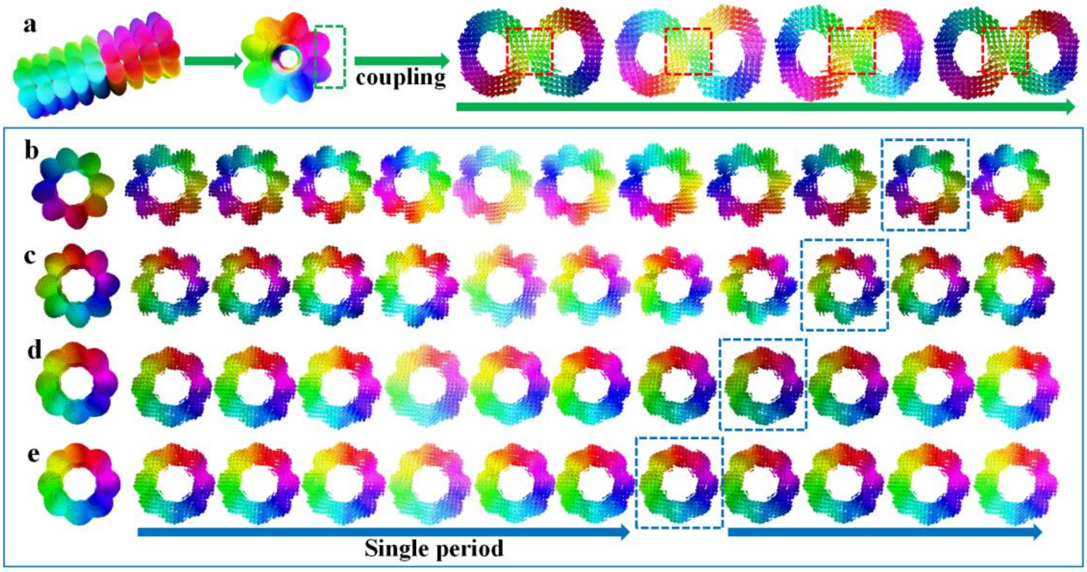

Figure S8. The micromagnetic simulation for a) the overall view of high-density nanospindle arrays and magnetic coupling of two neighbored spindles, b-e) the cross-section images of cyclical variation of magnetic moments under alternating magnetic fields with the increasing content of Fe component.

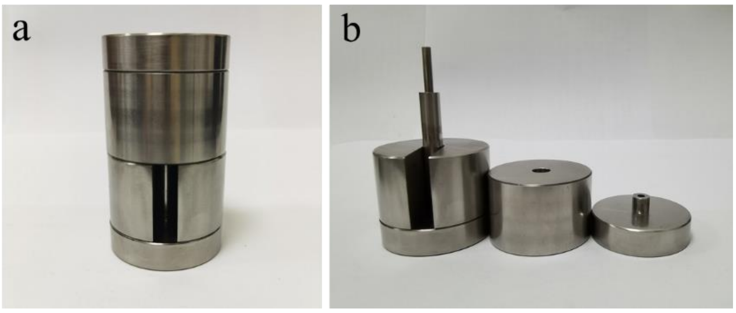

Figure S9. Digital photos of the experimental set-up for preparing the samples.

The explanation of electronic holography technology: Electronic holography technology can offer the information about the phase change after the incident electron wave passing through the materials, which is the main and important application. The interaction between the electron wave and the substance produces a phase change. Holography is the interference generated by the Fresnel diffraction

generated by the object and the coherent background. The resulting hologram is not like an object, but contains a reproducible object and the necessary amplitude and phase information. When the electron wave passes through the substance, it will be modulated by the potential field inside the material. The phase of the outgoing electron wave on the lower surface will be ahead of (or lag behind) the phase of the reference electron wave in the same direction. According to the Poisson's equation:

$$\rho(\chi) = -\varepsilon_\gamma \varepsilon_0 \frac{\partial^2 v(\chi)}{\partial \chi^2}$$

where $\rho(\chi)$ represents the charge density, ε_γ and ε_0 represent the relative dielectric constant of samples and vacuum, respectively. χ is the distance.

The phase diagram can be obtained by software processing to gain the internal potential distribution of the substance. In other word, through electronic holography, we can obtain the electric field and valence electron distribution inside the material. Further second-order differentiation of the phase diagram results in a charge density distribution of the selected/marked region.

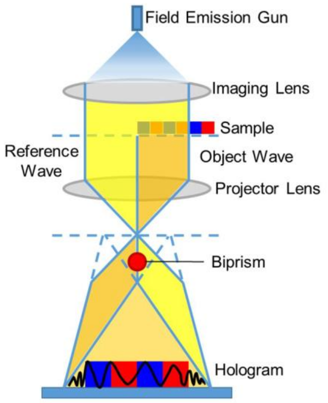

Figure S10. Schematic of the off-axis electron holography technology. By applying a voltage to the biprism below the sample, an interference pattern or hologram could be produced through the interference between the reference wave which passes through the vacuum and the objective wave which passes through the sample. After the reconstruction, the phase and amplitude components of the object wave could be

obtained.

Table S1. Microwave absorption performance of Fe-based absorbents and other magnetic materials in previous references and this work.

Absorbents	Thickness (mm)	Maximum RL (dB)	EAB (GHz)	Refs
NC@Co/N	2.2	-52.50	4.4	[1]
PAN/CNTs/Fe_3O_4	1.5	-59.85	4.0	[2]
Fe_3O_4/C	4.3	-43.95	3.0	[3]
C@$NiCo_2O_4$@Fe_3O_4	3.4	-43.0	2.1	[4]
Fe_3O_4@PB composite	1.55	-48.04	3.5	[5]
Fe/C	2.5	-29.50	3.1	[6]
Co-C/MWCNTs	1.8	-50.0	3.6	[7]
Fe_3O_4/C/PVDF	2.1	-38.8	2.6	[8]
Fe_3O_4/Fe nanoring	4.0	-23.09	3.9	[9]
Fe/Fe_3O_4@C	3.0	-55.4	4.2	This work

[1] P. Liu, S. Gao, Y. Wang, Y. Huang, W. He, W. Huang, J. Luo, *Chem. Eng. J.* **2020**, *381*, 122653.

[2] Y. Li, X. Liu, X. Nie, W. Yang, Y. Wang, R. Yu, J. Shui, *Adv. Funct. Mater.* **2019**, *29*, 1807624.

[3] Y. Liu, Y. Fu, L. Liu, W. Li, J. Guan, G. Tong, *ACS Appl. Mater. Inter.* **2018**, *10*, 16511.

[4] S. Wei, X. Wang, B. Zhang, M. Yu, Y. Zheng, Y. Wang, J. Liu, *Chem. Eng. J.* **2017**, *314*, 477.

[5] W. Liu, J. Liu, Z. Yang, G. Ji, *ACS Appl. Mater. Inter.* **2018**, *10*, 28887.

[6] Q. Liu, X. Liu, H. Feng, H. Shui, R. Yu, *Chem. Eng. J.* **2017**, *314*, 320.

[7] R. Shu, W. Li, Y. Wu, J. Zhang, G. Zhang, *Chem. Eng. J.* **2019**, *362*, 513.

[8] W. Xu, G.-S. Wang, P.-G. Yin, *Carbon* **2018**, *139*, 759.

[9] Y. Ding, L. Zhang, Q. Liao, G. Zhang, S. Liu, Y. Zhang, *Nano Res.* **2016**, *9*, 2018.
